# Genotype-phenotype correlations in Chinese von Hippel–Lindau disease patients

**DOI:** 10.18632/oncotarget.16594

**Published:** 2017-03-27

**Authors:** Shuanghe Peng, Matthew J. Shepard, Jiangyi Wang, Teng Li, Xianghui Ning, Lin Cai, Zhengping Zhuang, Kan Gong

**Affiliations:** ^1^ Department of Urology, Peking University First Hospital, Beijing, P.R China; ^2^ Institute of Urology, Peking University, Beijing, P.R China; ^3^ National Urological Cancer Center, Beijing, P.R China; ^4^ Department of Neurological Surgery, University of Virginia, Charlottesville, Virginia, USA; ^5^ Surgical Neurology Branch, National Institute of Neurological Disorders and Stroke, National Institutes of Health, Bethesda, Maryland, USA

**Keywords:** von Hippel–Lindau, VHL, genotype-phenotype correlation, VHL mutation, phenotypic predictor

## Abstract

von Hippel–Lindau (VHL) disease is caused by mutations in the *VHL* gene and demonstrates marked phenotypic variability. Genotype-phenotype correlations in Chinese VHL patients have been unclear. To establish genotype-phenotype correlations in Chinese VHL patients, we collected *VHL* mutations and phenotypes of 291 patients with VHL disease from 115 unrelated families. Genotype-phenotype correlations at mutation type level, mutation region level, and mutation codon level were analyzed by Kaplan-Meier curves and Cox regression models. We found missense mutations conferred an increased risk of pheochromocytoma developments, but a decreased risk of central nervous system hemangioblastomas (CHBs) and pancreatic lesions. Patients with *VHL* deletions were more prone to developing retinal angiomas. Renal cell carcinomas were more frequent in nonsense, frameshift or splice-site mutations. Mutations in Exon 2 conferred a higher risk and earlier diagnostic age of CHBs than mutations in other exons (HR = 1.684, 95% CI 1.082–2.620, *p* = 0.021; 27.0 ± 9.7 years versus 32.8 ± 11.7 years, *p* = 0.024), while patients with mutations in Exon 3 were more prone to developing pheochromocytomas (HR = 2.760, 95% CI 1.419–5.370, p = 0.003). Mutations at codon 80 or codon167 conferred significantly higher risks of pheochromocytomas than other mutations (HR = 4.678, 95% CI 1.392–15.724, *p* = 0.013; HR = 4.683, 95% CI 2.515–8.719, *p* < 0.001 respectively). In conclusion, VHL mutation types, mutation regions and mutation codons can act as phenotypic predictors of VHL disease. Mutation regions and mutation codons may aid in directed surveillance and monitoring of VHL patients.

## INTRODUCTION

von Hippel–Lindau disease (VHL; OMIM Number 193300) is an autosomal-dominant inherited tumor predisposition syndrome. Patients with VHL are prone to the development of multiple neoplastic lesions including central nervous system hemangioblastomas (CHBs), retinal angiomas (RAs), renal cell carcinomas (RCCs), renal cysts, pancreatic tumors or cysts (PCTs), pheochromocytomas (PHEOs), endolymphatic-sac tumors, and papillary cystadenomas of the epididymis or broad ligament [1–6]. The incidence of VHL disease is approximately 1 in 36000 live births and the overall penetrance is greater than 90% by age 65 [1].

VHL disease is caused by germline mutations in the *VHL* tumor suppressor gene, which is located on chromosome 3p25-26 [7]. The gene consists of 3 exons: exon 1 spans codons 1–113 (nucleotides 1–340), exon 2 spans codons 114–154 (nucleotides 341–463), and exon 3 spans codons 155–213 (nucleotides 464–642) [7–9]. The protein product of the *VHL* gene (pVHL) has two main domains. One b-sheet domain (residues 63–154) binds the a subunits of hypoxia inducible factor (HIF) at residues 65–117 and one a-helical domain (residues 155–192) binds Elongin B, Elongin C and Cul2 at residues 158–184 thereby forming the VCB-Cul2 complex [10–12]. pVHL plays a key role in regulating proteolytic degradation of the a subunits of HIF in normoxic conditions [13–15]. In states of normoxia, the VCB-Cul2 complex leads to the ubiquitination of HIF thereby promoting its degradation in the proteasome [2, 16]. pVHL inactivation leads to HIF stabilization and activation of downstream target genes that are overexpressed in the highly vascularized tumors that are characteristic of VHL disease [17, 18].

The clinical diagnosis of VHL disease can be made if a single VHL associated tumor (hemangioblastoma, PHEO or RCC) occurs in a patient with a family history of VHL [4]. In the absence of a positive family history of the disorder, the diagnosis can also be made if a patient harbors two or more hemangioblastomas or both a hemangioblastoma and a relative visceral tumor [6, 19]. Patients can also be diagnosed with VHL disease if germline mutations of the *VHL* gene have been identified. The detection of *VHL* mutations aids in the early diagnosis of the disease for at risk individuals, but also enables the study of genotype-phenotype correlations.

VHL disease demonstrates marked phenotypic variability, and patients may develop manifestations from early childhood and through adulthood [20, 21]. The genotype-phenotype correlation in VHL disease has been examined previously and classification schemes have been proposed based on patient's predilections for PHEO development. VHL Type 1 is characterized by truncating mutations and confers a low risk for PHEOs while VHL Type 2 is characterized by missense mutations (M mutations) which are associated with an increased risk for PHEOs [22–26]. Type 2 was subsequently divided into Type 2A (hemangioblastoma and PHEO but rarely RCC), Type 2B (susceptible to hemangioblastoma, RCC, and PHEO), and Type 2C (PHEO only) [27, 28]. These correlations have provided useful strategies for prophylactic surveillance and genetic counseling of asymptomatic members in VHL families. These existing studies are mainly derived from western countries and the genotype-phenotype correlations from Chinese VHL cohorts remain unclear. Furthermore, the current VHL classification schemes are imperfect and up to 44% of patients with VHL Type 1 will harbor a M mutation while 15% of Type 2 patients will not harbor a truncating VHL mutation [3]. Thus, a better understanding of specific genotype-phenotype correlations is imperative for targeted surveillance and counseling of VHL disease which bears the shortest median survival of all tumor predisposition syndromes [29].

Herein, we analyze a large number of VHL patients to date in order to: (1) analyze how different mutation types of the *VHL* gene have influenced phenotypes in VHL patients, (2) assess the correlations between mutation regions and phenotypes, and (3) establish genotype-phenotype correlations of the disease at codon level.

## RESULTS

### *VHL* mutations

**Figure 1:**
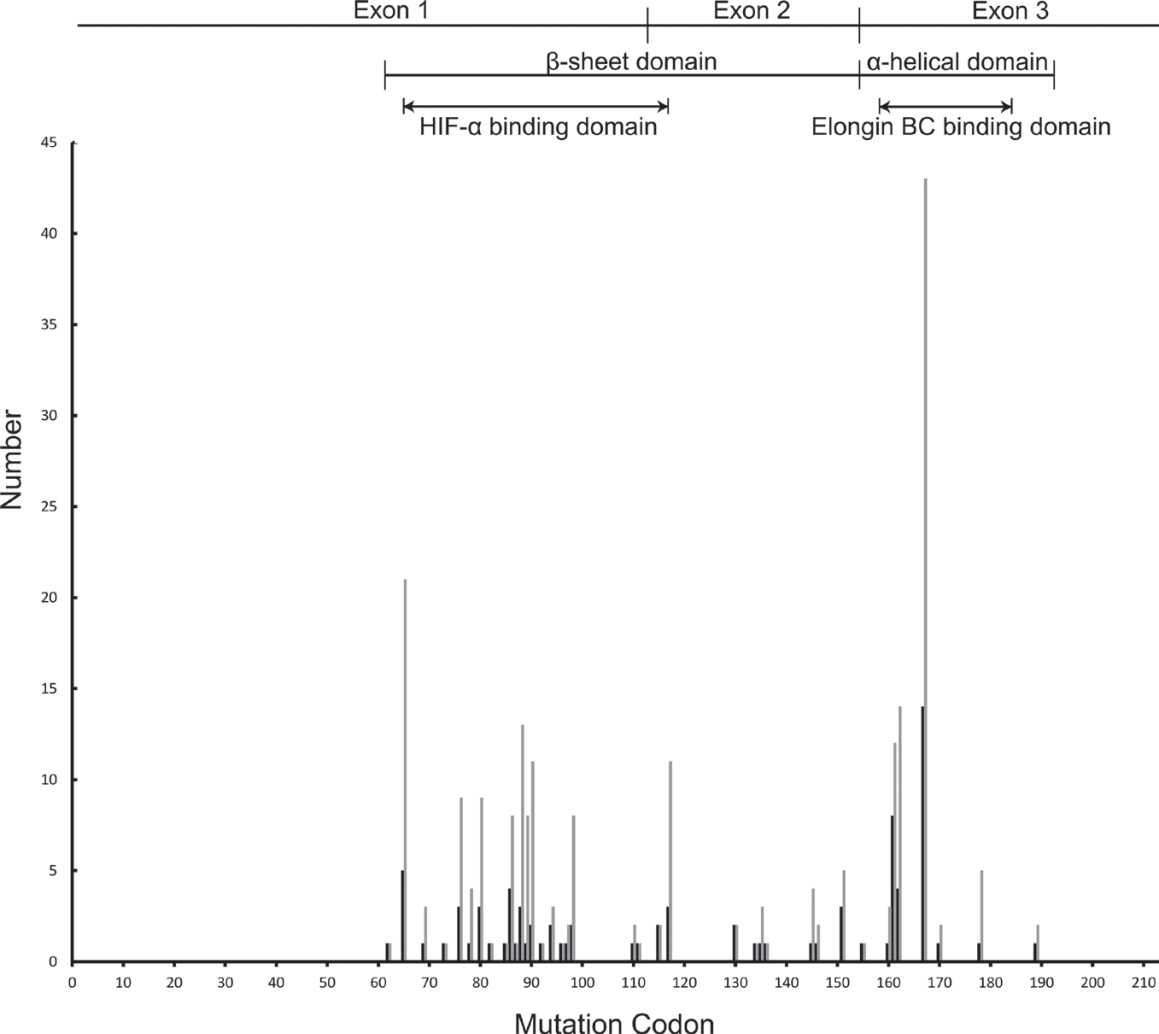
The distribution of germline mutations by codon in Chinese VHL families and patients The dark lines on the Y-axis represent mutational family numbers and the light lines represent mutational patient numbers. All intragenic mutations were located between codons 62 and 189. The most commonly mutated codon was codon 167.

A total of 61 different intragenic *VHL* mutations were identified within 89 unrelated families: 31 missense (50 families, 156 patients), 7 nonsense (15 families, 25 patients), 17 micro-deletions or micro-insertions (18 families, 41 patients), 6 splice-site mutations (6 families, 15 patients). Large deletions of the *VHL* gene (DEL mutations) were detected in 54 patients from 26 families. Nonsense mutations, small deletions and insertions, and splice site mutations were categorized as “NSS” mutations. There were a total of 81 patients from 39 unrelated families had NSS mutations. The distribution of germline mutations by codon in studied families and patients is shown in Figure 1.

### Clinical features of VHL disease

**Figure 2:**
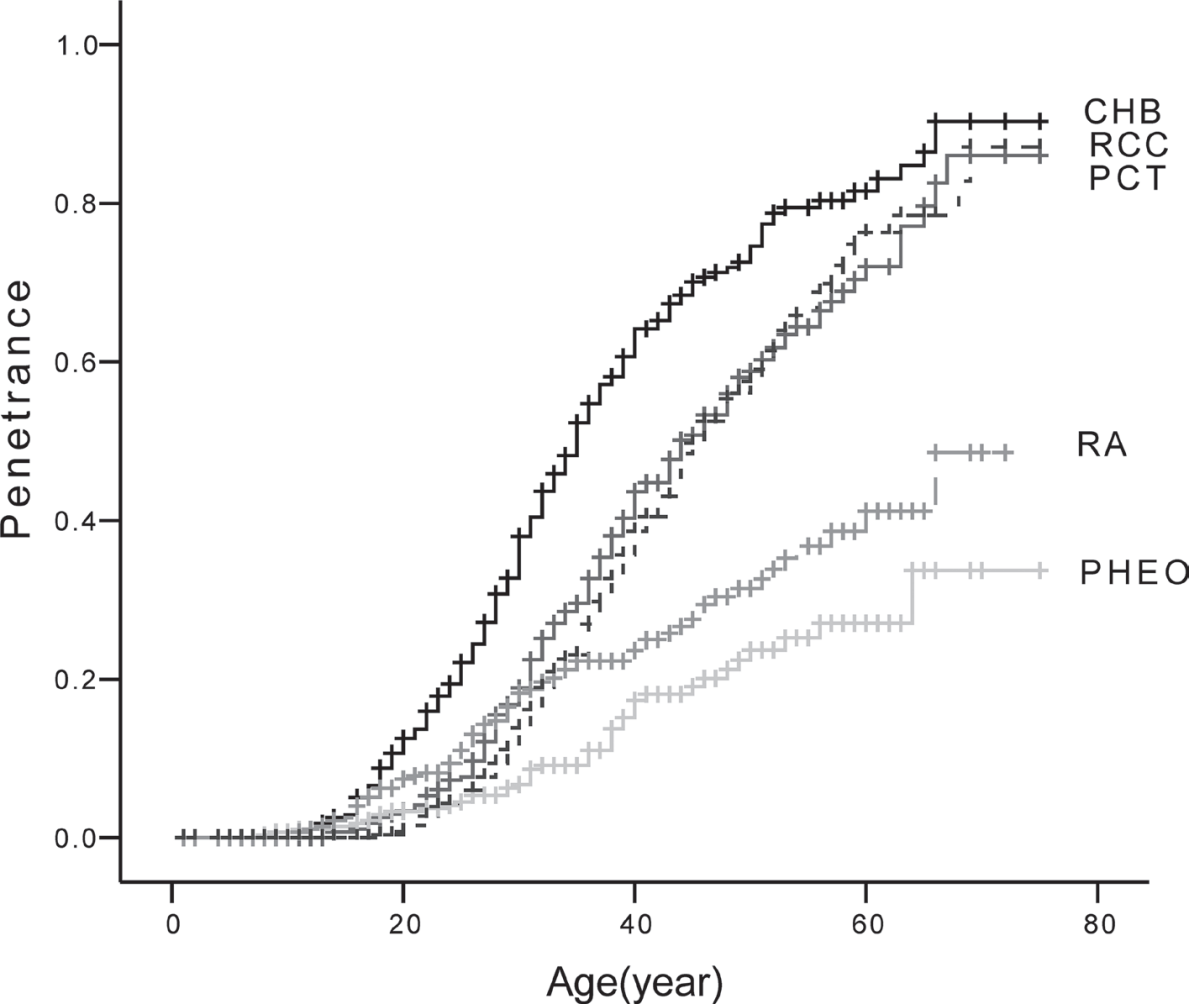
Age-related risks of the five major VHL lesions Abbreviations: CHB, central nervous system hemangioblastoma; RA, retinal angioma; RCC, renal cell carcinoma; PCT, pancreatic cyst or tumor; PHEO, pheochromocytoma.

The prevalence of the five major VHL lesions were: CHB 64.6% (*n* = 188), RA 24.7% (*n* = 72), RCC 44.3% (*n* = 129), PCT 47.4% (*n* = 138), and PHEO 14.8% (*n* = 43). The mean diagnostic age for each lesion was as follows: CHB 31.4 ± 11.4 years, RA 29.9 ± 12.9 years, RCC 38.8 ± 11.0 years, PCT 36.3 ± 11.7 years, and PHEO 32.7 ± 12.9 years. Age-related risks for the five major lesions are presented in Figure 2. The genotype and phenotype of each patient are shown in Supplementary Table 1.

The influence of sex on the onset risk and survival of studied patients had been assessed, and no difference was found between female and male patients (HR = 0.960, 95% CI 0.753–1.225, *p* = 0.744; HR = 1.035, 95% CI 0.653–1.641, *p* = 0.885 respectively).

### Genotype-phenotype correlations

#### Mutation types and phenotypes

**Figure 3:**
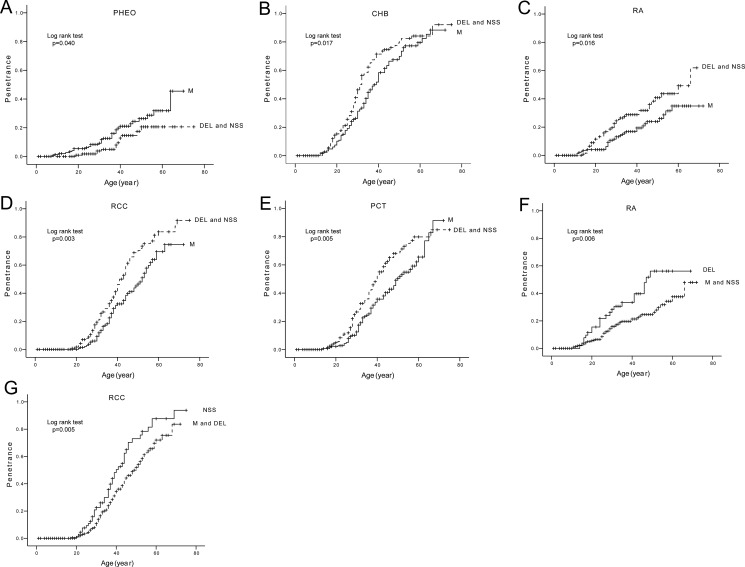
Comparison of age-related risks of major VHL lesions in mutation type level Comparison of age-related risks of PHEO (**A**), CHB (**B**), RA (**C**), RCC (**D**), and PCT (**E**) in VHL patients with missense mutations (M group) and those with other mutations (DEL and NSS group). Comparison of age-related risk of RA (**F**) in VHL patients with large deletions of *VHL* gene (DEL group) and those with other mutations (M and NSS group). Comparison of age-related risk of RCC (**G**) in VHL patients with nonsense, frameshift, and splice mutations (NSS group) and those with other mutations (M and DEL group).

We compared the age-related risks and mean diagnostic ages of the five major VHL lesions in patients with different mutation types (Table 1, Figure 3). M mutations conferred an increased risk of PHEOs (Figure 3A), but a decreased risk of CHBs, RAs, RCCs and PCTs as compared to DEL and NSS mutations (Figure 3B–3E). DEL mutations were more prone to developing RAs than M and NSS mutations (Figure 3F). RCCs were more frequent in the setting of NSS mutations compared to M and DEL mutations (Figure 3G). The mean diagnostic age of CHBs was significantly earlier in patients with DEL and NSS mutations than in patients with M mutations.

**Table 1:** Comparison of the age-related risks and mean diagnostic ages of the five major VHL lesions in patients with different mutation types

Group	Lesion	Age-related Risk			Mean Diagnostic Age	
		HR	95% CI	*p* Value	Mean ± SD (year)	*p* Value
DEL and NSS group vs M group	CHB	1.406	1.055–1.874	**0.020**	29.7 ± 10.6 vs 33.0 ± 11.9	**0.045**
	RA	1.762	1.105–2.811	**0.017**	29.0 ± 13.0 vs 31.0 ± 13.0	0.531
	RCC	1.671	1.179–2.368	**0.004**	37.8 ± 11.3 vs 39.9 ± 10.6	0.281
	PCT	1.595	1.140–2.230	**0.006**	34.6 ± 10.8 vs 38.4 ± 12.4	0.056
M group vs DEL and NSS group	PHEO	1.947	1.015–3.736	**0.045**	31.4 ± 14.2 vs 35.7 ± 9.2	0.328
DEL group vs M and NSS group	CHB	1.313	0.919–1.875	0.135	29.8 ± 10.0 vs 31.8 ± 11.7	0.329
	RA	1.993	1.206–3.291	**0.007**	28.6 ± 11.7 vs 30.4 ± 13.5	0.597
	RCC	1.166	0.762–1.783	0.479	38.2 ± 11.5 vs 38.9 ± 10.9	0.761
	PCT	1.352	0.901–2.029	0.146	33.3 ± 9.1 vs 37.2 ± 12.2	0.107
	PHEO	0.555	0.218–1.410	0.216	34.0 ± 10.1 vs 32.6 ± 13.4	0.817
NSS group vs M and DEL group	CHB	1.253	0.911–1.723	0.166	29.6 ± 11.2 vs 32.1 ± 11.4	0.178
	RA	1.087	0.643–1.836	0.756	29.5 ± 14.5 vs 30.0 ± 12.4	0.880
	RCC	1.666	1.153–2.407	**0.007**	37.6 ± 11.2 vs 39.4 ± 10.9	0.381
	PCT	1.409	0.982–2.021	0.063	35.4 ± 11.8 vs 36.8 ± 11.7	0.535
	PHEO	0.631	0.293–1.362	0.241	36.8 ± 9.2 vs 31.8 ± 13.6	0.335

Abbreviations: M, missense mutations; DEL, large deletions of the *VHL* gene; NSS, nonsense mutations, small deletions and insertions, and splice site mutations; CHB, central nervous system hemangioblastoma; RA, retinal angioma; RCC, renal cell carcinoma; PCT, pancreatic cyst or tumor; PHEO, pheochromocytoma; HR, hazard ration; SD, standard deviation.

#### Mutation regions and phenotypes

**Figure 4:**
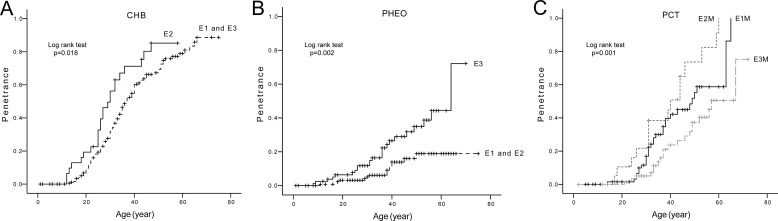
Comparison of age-related risks of major VHL lesions in mutation region level (**A**) Age-related risk of CHB in VHL patients with mutations in Exon 2 (E2 group) and those with mutations in Exon 1 and Exon 3 (E1 and E3 group). (**B**) Age-related risk of PHEO in VHL patients with mutations in Exon 3 (E3 group) and those with mutations in Exon 1 and Exon 2 (E1 and E2 group). (**C**) Age-related risk of PCT in VHL patients with missense mutations in Exon 1 (E1M group), Exon 2 (E2M group), and Exon 3 (E3M group).

The age-related risks and mean diagnostic ages of the five major VHL lesions in patients with mutations in different Exons of *VHL* gene were also analyzed. The results are presented in Table 2 and Figure 4 (parts of negative results not presented). Mutations in Exon 2 conferred a higher risk and a nearly 6 year earlier mean diagnostic age of CHBs compared to mutations in Exon 1 and Exon 3 (Figure 4A), while patients with mutations in Exon 3 were more prone to developing PHEOs than patients with mutations in Exon 1 or Exon 2 (Figure 4B). M mutations in Exon 1 and Exon 2 conferred higher risks of PCTs than M mutations in Exon 3. There was also a significant difference between the age-related risk curves of PCTs in patients with M mutations in Exon 1 and Exon 2 (*p* = 0.035) (Figure 4C). Individuals with M mutations in Exon 3 were more prone to developing PHEOs than their counterparts with M mutations in Exon 1.

**Table 2:** Comparison of the age-related risks and mean diagnostic ages of the five major VHL lesions in patients with mutations in different Exons of the *VHL* gene

Group	Lesion		Age-related Risk			Mean Diagnostic Age	
			HR	95% CI	*p* Value	Mean ± SD (year)	*p* Value
E2 group vs E1 and E3 group	CHB		1.684	1.082–2.620	**0.021**	27.0 ± 9.7 vs 32.8 ± 11.7	**0.024**
	RA		0.792	0.336–1.864	0.593	29.7 ± 12.4 vs 30.6 ± 13.9	0.882
	RCC		1.341	0.802–2.242	0.263	39.0 ± 10.8 vs 38.7 ± 10.7	0.897
	PCT		1.52	0.938–2.463	0.089	37.1 ± 12.5 vs 37.1 ± 12.1	0.988
	PHEO		0.882	0.342–2.269	0.794	28.8 ± 10.5 vs 33.0 ± 14.0	0.529
E3 group vs E1 and E2 group	CHB		0.721	0.508–1.023	0.067	32.6 ± 11.6 vs 31.5 ± 11.6	0.590
	RA		0.713	0.309–1.303	0.272	29.9 ± 12.8 vs 30.7 ± 14.2	0.839
	RCC		0.823	0.544–1.245	0.356	38.8 ± 10.9 vs 38.7 ± 10.6	0.975
	PCT		0.732	0.486–1.102	0.134	37.2 ± 12.2 vs 37.0 ± 12.2	0.933
	PHEO		2.760	1.419–5.370	**0.003**	32.7 ± 14.8 vs 31.9 ± 11.6	0.851
E1M group vs E2M group vs E3M group	CHB	E1M vs E3M	1366	0.884–2.110	0.160	34.0 ± 11.9 vs 27.7 ± 12.0 vs 33.9 ± 11.7	0.195
		E2M vs E3M	1.762	0.947–3.280	0.074		
	RA	E1M vs E3M	1.234	0.569–2.675	0.595	30.8 ± 12.8 vs 30.6 ± 13.6 vs 31.3 ± 14.0	0.992
		E2M vs E3M	1.487	0.523–4.229	0.457		
	RCC	E1M vs E3M	1.139	0.649–2.000	0.650	40.6 ± 10.3 vs 39.2 ± 10.7 vs 39.5 ± 11.3	0.919
		E2M vs E3M	1.821	0.869–3.818	0.112		
	PCT	E1M vs E3M	1.898	1.064–3.383	**0.030**	37.0 ± 12.2 vs 37.5 ± 13.7 vs 41.0 ± 11.9	0.521
		E2M vs E3M	3.530	1.777–7.011	**< 0.001**		
	PHEO	E1M vs E3M	0.214	0.081–0.565	0.002	33.0 ± 13.3 vs 22.0 ± 7.0 vs 32.4 ± 15.0	0.494
		E2M vs E3M	0.470	0.140–1.576	0.221		

Abbreviations: E, exon; E1M, missense mutations in Exon 1; E2M, missense mutations in Exon 2; E3M, missense mutations in Exon 3; CHB, central nervous system hemangioblastoma; RA, retinal angioma; RCC, renal cell carcinoma; PCT, pancreatic cyst or tumor; PHEO, pheochromocytoma; HR, hazard ration; SD, standard deviation.

#### Mutation codons and phenotypes

**Figure 5:**
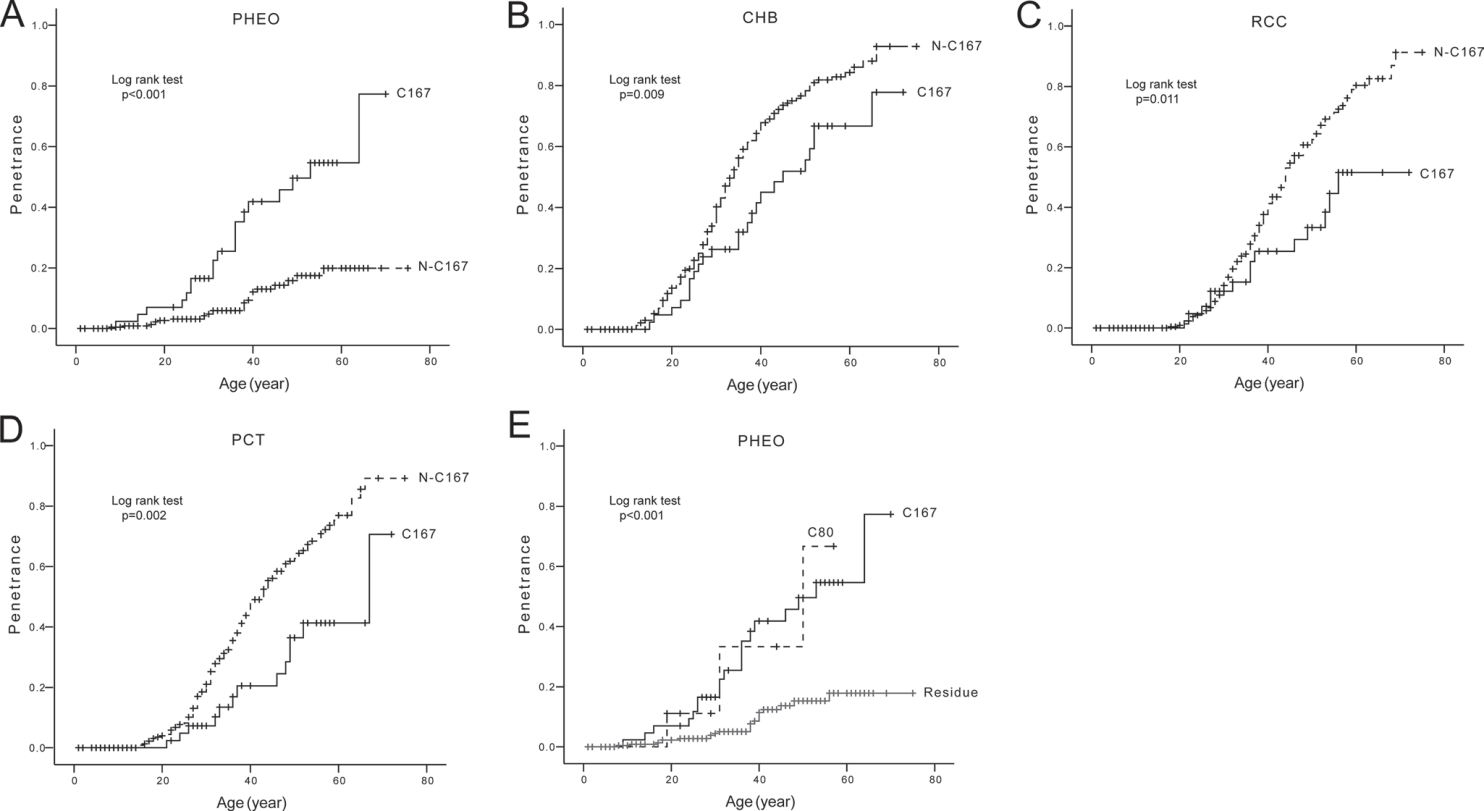
Comparison of age-related risks of major VHL lesions in mutation codon level Comparison of age-related risks of PHEO (**A**), CHB (**B**), RCC (**C**), and PCT (**D**) in VHL patients with mutations at codon 167 (C167 group) and those with other mutations (N-C167 group). Comparison of age-related risk of PHEO in patients with mutations at codon 80 (C80 group), mutations at codon 167 (C167 group), and other mutations (Residue group) (**E**).

Codon 167 was the most frequently mutated codon of *VHL* gene in our study. We compared the age-related risks and mean diagnostic ages of the five major VHL lesions in patients with mutations at codon 167 (C167 group) and patients with other mutations (N-C167 group). The results are summarized in Table 3 and Figure 5. C167 mutations conferred a significantly higher risk of PHEOs development, but a lower risk of CHBs, RCCs and PCTs compared to the N-C167 group (Figure 5A–5D). The risks of RAs in the two groups were similar.

**Table 3:** Comparison of the age-related risks and mean diagnostic ages of the five major VHL lesions in patients with mutations at codon 167 (C167 group) and patients with other mutations (N-C167 group)

Group	Lesion	Age-related Risk			Mean Diagnostic Age	
		HR	95% CI	*p* Value	Mean ± SD (year)	*p* Value
C167 group vs N-C167 group	CHB	0.572	0.372–0.880	**0.011**	34.8 ± 13.1 vs 31.0 ± 11.1	0.127
	RA	0.487	0.223–1.064	0.071	24.1 ± 7.9 vs 30.5 ± 13.2	0.219
	RCC	0.499	0.286–0.870	**0.014**	37.2 ± 12.3 vs 39.0 ± 10.8	0.579
	PCT	0.417	0.235–0.740	**0.003**	40.0 ± 13.2 vs 36.0 ± 11.5	0.237
	PHEO	4.223	2.311–7.716	**< 0.001**	33.2 ± 13.6 vs 32.3 ± 12.6	0.828

Abbreviations: CHB, central nervous system hemangioblastoma; RA, retinal angioma; RCC, renal cell carcinoma; PCT, pancreatic cyst or tumor; PHEO, pheochromocytoma; HR, hazard ration; SD, standard deviation.

From the distribution of germline mutations by codon in VHL patients, we found mutations at codon 80 (C80) and C167 conferred a higher risk of PHEOs than other mutations (Residue) (Figure 5E). This was statistically significant for both the C80 and C167 groups (HR = 4.678, 95% CI 1.392–15.724, *p* = 0.013; HR = 4.683, 95% CI 2.515–8.719, p < 0.001 respectively). The C80 group and C167 group had a similar predilection for PHEO development (*p* = 0.879).

### Genotype and Survival

We calculated age-related survival of patients with different *VHL* mutations and the results are presented in Supplementary Table 2. Median follow up was 5 years (range 1–42 years), over total 2305 person-years. Median expected survival of all studied patients was 60 years. Mean age at death was 42.6 ± 13.8 years. There was no significant difference between the risks of VHL-related death of patients with different *VHL* mutations.

## DISCUSSION

This study analyzed the largest cohort of Chinese VHL patients reported to date to investigate genotype-phenotype correlations in VHL disease. The results of our study indicate that mutation regions and mutation codons act as VHL phenotypic predictors and may aid in the directed surveillance and monitoring of VHL patients.

The understanding that M mutations confer an increased risk of PHEOs was the earliest recognized and most widely tested genotype-phenotype correlation in VHL patients from foreign countries [24–26]. These previous results are similar to our findings that M mutations confer a high risk of PHEO development in Chinese VHL patients. In addition, we report that mutations in Exon 3 were associated with an increased risk of PHEOs compared to mutations in Exon 1 and Exon 2. Likewise, M mutations in Exon 3 were associated with a greater risk of PHEOs than M mutations in Exon 1. This is consistent with the findings of Forman et al. who reported in 2009 that mutations in the Elongin C binding domain of pVHL (situated in Exon 3) are associated with PHEOs [30]. This domain has been implicated to be involved in p53 mediated apoptosis and thus it has been hypothesized that mutations of this domain may lead to PHEOs via p53 dysregulation [30, 31]. The majority of the mutations in Exon 3 in our study are situated in the Elongin C binding domain providing further clinical evidence that this region is imperative for PHEO development in VHL patients. Some mutations not located in this domain might still disrupt the interaction between pVHL and p53 [30]. This theory may be the reason for codon 80 mutations conferring a higher risk of PHEOs in our study.

Next, we further explored the genotype-phenotype correlations for PHEOs at the codon level and found mutations at codon 80 or codon167 conferred significantly higher risks of PHEOs than other mutations. Codon 167 has been implicated with PHEO development in VHL patients from foreign countries [24]. This study reveals Codon 80 confers a higher risk of PHEOs for the first time in Chinese patients. These results were inspiring because they indicated that mutation regions and mutation codons could act as phenotypic predictors for the presence of PHEOs in VHL patients. Further studies based on larger, multinational cohorts are needed to verify our results and to explore more mutation regions and mutation codons to act as VHL phenotypic predictors.

The pathogenesis of PHEOs remains unclear. It has been previously shown that M mutations on the surface of pVHL confer the highest rate of PHEO development while mutations within the protein or DEL mutations have low rates of PHEO development [26]. Thus it has been hypothesized that the mutations accountable for PHEO development may induce gain-of-function through an intact, but altered pVHL [11, 32, 33]. This is consistent with the finding that in patients with Type 2C VHL, pVHL remains able to downregulate HIF, thereby explaining the absence of RCC and CHB [12, 32, 33]. In our work, we found mutations at codon 167 conferred a much higher risk of PHEOs, but a lower risk of CHBs, RCCs and PCTs than other mutations. Mutant *VHL* gene carrying mutations at codon 167 could still be able to downregulate HIF and may account for the lower risk of CHBs, RCCs and PCTs in patients with mutations at this codon.

Hemangioblastomas including CHBs and RAs are important manifestations of VHL disease. Researchers from other countries have reported that DEL mutations and protein truncating mutations conferred a higher risk of CHBs than M mutations [24, 34, 35]. Frank et al. found DEL mutations were associated with an increased risk of RAs [34]. In our work, non-missense mutations had a higher risk and a nearly 3 year earlier diagnostic age of CHBs than M mutations. Patients with DEL mutations were more prone to developing RAs than other mutations. In addition to descriptive mutations, we also found that mutations in Exon 2 conferred a higher risk and a nearly 6 year earlier diagnostic age of CHBs than mutations in Exon 1 and Exon 3. Hemangioblastoma pathogenesis is believed to be secondary to dysregulation of the HIF pathway with inappropriate elevation of HIF-1a leading to increased expression of erythropoietin and vascular endothelial growth factor [36, 37]. Lee et al. had also reported that HIF-a binding site missense mutations elevated age-specific risk for CHB in Korean VHL patients [38]. Clifford et al. had theorized that both HIF deregulation and the loss of fibronectin binding were associated with an increased risk of hemangioblastomas [39]. DEL mutations lead to complete loss of function of VHL, however M mutations have been shown to affect pVHL stability without affecting its activity [40]. Thus, we hypothesize that hemangioblastoma formation is a result of aberrant HIF signaling secondary to a quantitative loss of pVHL.

In our study, we found NSS mutations conferred a higher risk of RCCs than DEL and M mutations. Ong et al. had also reported that nonsense and frameshift mutations had a higher age-related risk of RCCs and hemangioblastomas than M mutations in VHL patients from the United Kingdom [26]. Gallou et al. had found mutations located in Missense Cluster Regions MCR-1 (residues 74–90) and MCR-2 (residues 130–136) were associated with an increased risk of RCCs in French VHL patients [41]. However, no difference in RCC risk between M mutations within and outside these MCR regions was found in our work nor the study of Ong et al. Some authors have proposed that mutations causing HIF deregulation led to the pathogenesis of RCCs in VHL disease [30] while others have theorized that mutations in the Elongin C binding domain might disrupt p53 binding causing apoptosis suppression and RCC development [31]. It has not been clear why NSS mutations confer a higher risk of RCCs than DEL or M mutations. NSS mutations may result in an aberrant gene product with a retained HIF binding site, but not a functional Elongin C binding site. Such a mutant protein may bind to HIF with a higher avidity compared to wild-type pVHL while simultaneously losing its ability to interact with p53 via Elongin C. This theory may explain the higher risk of RCCs in patients with NSS mutations.

We found non-missense mutations had a significantly higher risk of PCTs than M mutations. In M mutations, mutations in Exon 1 and Exon 2 conferred an increased risk of PCTs than mutations in Exon 3. We hypothesize that M mutations conferring a lower risk of PCTs may be secondary to these mutations leading to an intact, but altered pVHL which was capable of HIF suppression. M mutations in different Exons conferring different risks of PCTs may be due to the different extent of HIF suppression. Additional studies are needed to investigate this mechanism.

In conclusion, we have investigated genotype-phenotype correlations in a large cohort of Chinese patients with VHL disease and report that mutation types, mutation regions and specific mutated codons serve as VHL phenotypic predictors. These results may prove useful for genetic counseling and targeted screening of patients with VHL disease. Additionally, these results provide a framework moving forward for future investigation into genotype-phenotype correlations for VHL and other tumor predisposition syndromes.

## MATERIALS AND METHODS

### Patient ascertainment and assessment

In this retrospective cohort study, we included all the VHL patients diagnosed at Peking University First Hospital (Beijing, China) prior to June 1 2016. A total of 291 patients from 115 different families were included for analysis. Individuals were diagnosed as patients with VHL if they carried a *VHL* germline mutation (*n* = 198) and/or fulfilled the clinical VHL criteria (*n* = 255) [4, 6, 19]. *VHL* mutation confirmation was verified in at least one patient of each family. The VHL status of family members who had not been verified by genetic testing was based on clinical criteria as obtained through interviews with patients, family members and medical record review. Of the included patients, 90.7% (264 of 291) harbored at least one VHL manifestation while the rest were asymptomatic mutation carriers. Follow up was determined from the time of first clinical presentation confirmed on imaging for symptomatic patients or the time of genetic diagnosis of VHL for asymptomatic mutation carriers to death or June 1 2016. Medical charts, radiographic and histological records on all affected individuals and on asymptomatic individuals who were proven *VHL* mutation carriers were subsequently reviewed to determine age at first diagnosis of the five major VHL lesions: CHB, RA, RCC (RCC or combined RCC and renal cysts), PCT (pancreatic cysts or combined cysts and tumors), and PHEO.

This project was approved by the Medical Ethics Committee of Peking University First Hospital (Beijing, China) and informed consent was obtained from patients or legal guardians.

### Molecular genetic analysis

Analysis for mutations of the *VHL* gene was performed on peripheral blood samples from all members of each kindred except members who had refused to the genetic testing or who had expired. *VHL* mutation analysis of the coding sequence and flanking intronic sequences was performed by PCR-direct sequencing. DEL mutations were detected by universal primer quantitative fluorescent multiplex PCR (UPQFMPCR). The primers and conditions for amplification have been described in our previous publication [42]. Missense mutations (leading to a single amino acid change in pVHL) were designated “M” mutations and large genomic deletions of the *VHL* gene were defined as “DEL” mutations. Other mutations including nonsense mutations (predicted to cause a truncated protein), small deletions and insertions (causing a frameshift), and splice site mutations were categorized as “NSS” mutations. Mutations in Exon 1 of the *VHL* gene were designated E1 mutations, mutations in Exon 2 were designated E2 mutations, and mutations in Exon 3 were designated E3 mutations. Missense mutations in Exon 1 were defined as E1M mutations, missense mutations in Exon 2 were defined as E2M mutations, and missense mutations in Exon 3 were defined as E3M mutations.

### Statistical analysis

Age-related risks of the five major VHL lesions were calculated using Kaplan-Meier plots and log-rank analysis. The effect of genotype on the risk of the five major VHL lesions was assessed in a Cox regression model. Statistical analyses were performed using SPSS 13.0. Statistical significance was taken at 5%.

## SUPPLEMENTARY TABLES




