# Supplementary material for: Genotype-phenotype correlations in Chinese von Hippel–Lindau disease patients
Source: Oncotarget. 2017 Mar 27;8(24):38456–65. doi: 10.18632/oncotarget.16594 (PMC5503545; doi:10.18632/oncotarget.16594)
Supplement: Supplementary file 1 [file oncotarget-08-38456-s001.pdf]

Genotype-phenotype correlations in Chinese von Hippel–Lindau disease patients

Supplementary Materials

Supplementary Table 1: The genotype and phenotype of Chinese von Hippel–Lindau disease patients. See Supplementary_Table_1

Supplementary Table 2: Comparison of the age-related survival, median survival time, and mean age at death between patients with different mutation types

Group		Age-related Survival			Median Survival Time		Mean Age at Death	
		HR	95% CI	*p* value	Median (year)	*p* Value	Mean ± SD (year)	*p* Value
M group vs DEL and NSS group		1.101	0.691–1.755	0.686	62 vs 58	0.683	43.0 ± 13.4 vs 42.0 ± 14.7	0.781
DEL group vs M and NSS group		0.895	0.481–1.665	0.726	60 vs 57	0.724	43.9 ± 14.6 vs 42.3 ± 13.8	0.718
NSS group vs M and DEL group		0.959	0.563–1.635	0.879	57 vs 60	0.877	40.8 ± 15.0 vs 43.2 ± 13.5	0.529
E2 group vs E1 and E3 group		0.892	0.422–1.886	0.765	60 vs 57	0.763	47.6 ± 15.7 vs 41.8 ± 13.6	0.273
E3 group vs E1 and E2 group		0.768	0.451–1.308	0.331	62 vs 57	0.324	44.6 ± 15.0 vs 41.5 ± 13.4	0.417
C167 group vs N-C167 group		0.865	0.473–1.583	0.637	66 vs 60	0.634	46.3 ± 15.3 vs 41.8 ± 13.5	0.288
E1M group vs E2M group vs E3M group	E1M vs E3M	1.884	0.980–3.620	0.058	53 vs 62 vs 62	0.127	39.3 ± 12.6 vs 49.3 ± 8.7 vs 46.4 ± 14.5	0.157
	E2M vs E3M	1.102	0.364–3.338	0.864				

Abbreviations: M, missense mutations; DEL, large deletions of the *VHL* gene; NSS, nonsense mutations, small deletions and insertions, and splice site mutations; E, exon; E1M, missense mutations in Exon 1; E2M, missense mutations in Exon 2; E3M, missense mutations in Exon 3; C176, mutations at codon 167; N-167, other mutations; HR, hazard ration; SD, standard deviation.
