# Supplementary material for: Genotype-phenotype correlations in Chinese von Hippel–Lindau disease patients
Source: Oncotarget. 2017 Mar 27;8(24):38456–65. doi: 10.18632/oncotarget.16594 (PMC5503545; doi:10.18632/oncotarget.16594)
Supplement: Supplementary file 2 [file oncotarget-08-38456-s002.docx]

| **Supplementary Table 1: The genotype and phenotype of Chinese von Hippel－Lindau disease patients** | | | | | | | | |
| --- | --- | --- | --- | --- | --- | --- | --- | --- |
| **Family Number** | **Patient Number** | **Patient** | **Nucleotide and Protein Change** | **First diagnostic age (year)** | | | | |
|  |  |  |  | **CHB** | **RA** | **RCC** | **PCT** | **PHEO** |
| 1 | 1 | Proband, M | c.185_193delTGCTGCGCTinsAGCA p.Val62Glufs*71 | 35 | － | 36 | 36 | － |
| 2 | 2 | Proband, M | c.194C>G p.Ser65Trp | － | － | 48 | 48 | － |
|  | 3 | Son | c.194C>G p.Ser65Trp | 22 | － | － | － | － |
|  | 4 | Mother | Unknown^a^ | 61 | － | － | － | － |
|  | 5 | Brother | Unknown^b^ | 33 | － | － | － | － |
| 3 | 6 | Proband, F | c.194C>G p.Ser65Trp | 34 | － | 37 | 37 | － |
| 4 | 7 | Proband, M | c.194C>T p.Ser65Leu | － | － | 33 | 33 | － |
| 5 | 8 | Proband, M | c.194C>T p.Ser65Leu | 21 | － | － | 30 | － |
|  | 9 | Grandfather | Unknown^a^ | 30 | － | － | － | － |
|  | 10 | Father | Unknown^a^ | 36 | － | － | － | － |
|  | 11 | Uncle | Unknown^a^ | 33 | － | － | － | － |
| 6 | 12 | Proband, F | c.194C>T p.Ser65Leu | － | － | 36 | 37 | － |
|  | 13 | Daughter | c.194C>T p.Ser65Leu | － | － | － | － | － |
|  | 14 | Mother | c.194C>T p.Ser65Leu | 59 | － | － | － | － |
|  | 15 | Uncle1 | c.194C>T p.Ser65Leu | 45 | － | 59 | 65 | － |
|  | 16 | Son of Uncle1 | Unknown^a^ | 22 | － | － | － | － |
|  | 17 | Uncle2 | Unknown^a^ | 51 | － | 51 | 51 | － |
|  | 18 | The first son of Uncle2 | c.194C>T p.Ser65Leu | 22 | － | 30 | 31 | － |
|  | 19 | Granddaughter of Uncle2 | c.194C>T p.Ser65Leu | － | － | － | － | － |
|  | 20 | The second son of Uncle2 | c.194C>T p.Ser65Leu | － | － | － | － | － |
|  | 21 | Aunt | c.194C>T p.Ser65Leu | 53 | 53 | － | － | － |
|  | 22 | Uncle3 | c.194C>T p.Ser65Leu | － | － | － | 49 | － |
| 7 | 23 | Proband, M | c.204_205insG p.Arg69Alafs*63 | 31 | － | － | － | － |
|  | 24 | Brother | c.204_205insG p.Arg69Alafs*63 | 32 | － | 32 | 32 | － |
|  | 25 | Father | c.204_205insG p.Arg69Alafs*63 | － | － | 58 | 58 | － |
| 8 | 26 | Proband, M | c.217C>T p.Gln73* | 66 | 66 | 37 | 54 | － |
| 9 | 27 | Proband, F | c.227-229delTCT p.Phe76del | 36 | － | 40 | 40 | － |
| 10 | 28 | Proband, M | c.224-226delTCT p.Phe76Cysfs*138 | 21 | － | － | － | － |
|  | 29 | Son | c.224-226delTCT p.Phe76Cysfs*138 | － | － | － | － | － |
| 11 | 30 | Proband, F | c.224-226delTCT p.Phe76Cysfs*138 | 28 | 26 | 37 | 28 | － |
|  | 31 | Son | c.224-226delTCT p.Phe76Cysfs*138 | － | － | － | － | － |
|  | 32 | Father | Unknown^a^ | 42 | － | － | － | － |
|  | 33 | Brother1 | Unknown^a^ | 28 | － | － | － | － |
|  | 34 | Brother2 | Unknown^a^ | 30 | － | － | － | － |
|  | 35 | Uncle | Unknown^a^ | － | 60 | - | - | － |
| 12 | 36 | Proband, M | c.233A>G p.Asn78Ser | 34 | － | 34 | 34 | － |
|  | 37 | Sister | c.233A>G p.Asn78Ser | － | － | - | 27 | － |
|  | 38 | Father | c.233A>G p.Asn78Ser | － | － | 55 | － | － |
|  | 39 | Uncle | Unknown^a^ | 19 | － | － | － | － |
| 13 | 40 | Proband, M | c.239G>T p.Ser80Ile | － | 33 | 31 | 31 | 31 |
| 14 | 41 | Proband, F | c.239delG p.Ser80Ilefs*79 | － | － | 44 | 44 | - |
| 15 | 42 | Proband, M | c.239_245delGTCCGCG p.Ser80Thrfs*77 | 32 | － | 32 | 52 | 50 |
|  | 43 | Daughter | c.239_245delGTCCGCG p.Ser80Thrfs*77 | － | 17 | 21 | 18 | 19 |
|  | 44 | Son | c.239_245delGTCCGCG p.Ser80Thrfs*77 | － | － | － | － | － |
|  | 45 | Sister | Unknown^a^ | 22 | － | － | － | － |
|  | 46 | Brother | Unknown^a^ | － | 52 | 52 | － | － |
|  | 47 | Niece1 | c.239_245delGTCCGCG p.Ser80Thrfs*77 | － | 22 | 22 | － | － |
|  | 48 | Niece2 | c.239_245delGTCCGCG p.Ser80Thrfs*77 | 19 | 18 | － | － | － |
| 16 | 49 | Proband, M | c.245G>C p.Arg82Pro | 40 | － | － | 26 | － |
| 17 | 50 | Proband, F | c.254T>C p.Leu85Pro | － | － | 30 | 30 | － |
| 18 | 51 | Proband, M | c.256C>T p.Pro86Ser | 31 | － | 32 | 32 | － |
|  | 52 | Father | Unknown^a^ | － | － | 52 | － | － |
|  | 53 | Daughter | c.256C>T p.Pro86Ser | － | 11 | － | － | － |
| 19 | 54 | Proband, F | c.256C>T p.Pro86Ser | － | 12 | 33 | 33 | － |
|  | 55 | Daughter | c.256C>T p.Pro86Ser | － | － | － | － | － |
| 20 | 56 | Proband, M | Unknown^a^ | 34 | 32 | 38 | 38 | － |
|  | 57 | Daughter | c.257C>T p.Pro86Leu | 18 | － | － | － | － |
| 21 | 58 | Proband, M | c.257C>G p.Pro86Arg | 26 | 26 | 26 | 26 | － |
| 22 | 59 | Proband, M | c.260T>A p.Val87Glu | － | － | － | 14 | 11 |
| 23 | 60 | Proband, M | c.263G>C p.Trp88Ser | 25 | 25 | 30 | 30 | － |
|  | 61 | Son | c.263G>C p.Trp88Ser | － | － | － | － | － |
|  | 62 | Daughter | c.263G>C p.Trp88Ser | － | － | － | － | － |
|  | 63 | Father | Unknown^a^ | 35 | 35 | － | － | － |
|  | 64 | Sister | Unknown^a^ | 24 | － | － | 31 | － |
|  | 65 | Aunt1 | Unknown^a^ | 35 | － | － | － | － |
|  | 66 | Aunt2 | Unknown^a^ | 20 | － | － | － | － |
|  | 67 | Aunt3 | Unknown^a^ | 44 | － | － | － | － |
|  | 68 | Son of Aunt3 | c.263G>C p.Trp88Ser | 26 | － | － | － | － |
| 24 | 69 | Proband, M | c.263G>A p.Trp88* | － | － | 34 | 38 | 39 |
|  | 70 | Mother | c.263G>A p.Trp88* | 34 | － | － | － | － |
| 25 | 71 | Proband, F | c.263G>A p.Trp88* | 30 | － | 28 | 28 | － |
|  | 72 | Son | c.263G>A p.Trp88* | － | － | － | － | － |
| 26 | 73 | Proband, M | c.266T>C p.Leu89Pro | 20 | － | － | 25 | － |
|  | 74 | Father | Unknown^a^ | 40 | 40 | － | － | － |
|  | 75 | Aunt1 | Unknown^a^ | 31 | － | － | － | － |
|  | 76 | The first son of Aunt1 | Unknown^b^ | － | － | 38 | － | － |
|  | 77 | Granddaughter of Aunt1 | Unknown^b^ | － | 16 | － | － | － |
|  | 78 | The second son of Aunt1 | Unknown^b^ | 30 | － | － | － | － |
|  | 79 | Uncle | Unknown^a^ | － | － | 52 | － | － |
|  | 80 | Aunt2 | Unknown^a^ | － | － | 45 | － | 45 |
| 27 | 81 | Proband, M | c.269A>T p.Asn90Ile | 28 | － | 38 | 38 | 38 |
|  | 82 | Mother | Unknown^a^ | 33 | － | － | － | － |
|  | 83 | Aunt1 | Unknown^a^ | 32 | － | － | － | － |
|  | 84 | Son of Aunt1 | Unknown^a^ | 30 | － | － | － | － |
|  | 85 | Aunt2 | c.269A>T p.Asn90Ile | － | － | － | 50 | － |
|  | 86 | Son of Aunt2 | c.269A>T p.Asn90Ile | － | － | － | － | － |
|  | 87 | Granddaughter of Aunt2 | c.269A>T p.Asn90Ile | － | － | － | － | － |
| 28 | 88 | Proband, M | c.269A>T p.Asn90Ile | 20 | 30 | － | － | － |
|  | 89 | Brother1 | c.269A>T p.Asn90Ile | 33 | － | － | － | － |
|  | 90 | Mother | c.269A>T p.Asn90Ile | 63 | － | 63 | 63 | － |
|  | 91 | Brother2 | Unknown^a^ | 16 | － | － | － | － |
| 29 | 92 | Proband, M | c.275delAinsCC p.Asp92Alafs*40 | 23 | － | － | 24 | － |
| 30 | 93 | Proband, M | c.280G>T p.Glu94* | 31 | － | － | － | － |
| 31 | 94 | Proband, M | c.280G>T p.Glu94* | 56 | － | 53 | 53 | － |
|  | 95 | Son | c.280G>T p.Glu94* | － | 29 | 29 | 29 | 29 |
| 32 | 96 | Proband, M | c.286C>T p.Gln96* | 16 | 16 | － | － | － |
| 33 | 97 | Proband, M | c.287_288insA p.Pro97Alafs*35 | 37 | 20 | 29 | 29 | － |
|  | 98 | Brother | c.287_288insA p.Pro97Alafs*35 | － | 32 | 43 | 43 | － |
| 34 | 99 | Proband, M | c.292T>A p.Tyr98Asn | 40 | 40 | 40 | 40 | 40 |
|  | 100 | Mother | Unknown^a^ | 50 | － | － | 63 | － |
|  | 101 | Sister1 | c.292T>A p.Tyr98Asn | 51 | 51 | － | － | － |
|  | 102 | Sister2 | Unknown^a^ | 40 | － | 43 | 43 | － |
|  | 103 | Brother | Unknown^a^ | 27 | 27 | － | － | － |
| 35 | 104 | Proband, F | c.293A>C p.Tyr98Ser | － | － | － | 27 | － |
|  | 105 | Sister | c.293A>C p.Tyr98Ser | － | － | － | 28 | － |
|  | 106 | Mother | Unknown^a^ | 45 | － | － | － | － |
| 36 | 107 | Proband, M | c.329delA p.His110Profs*49 | 18 | 18 | － | － | － |
|  | 108 | Mother | Unknown^a^ | 29 | 29 | － | － | － |
| 37 | 109 | Proband, M | c.332delG p.Ser111Thrfs*48 | 19 | － | 20 | 20 | － |
| 38 | 110 | Proband, M | c.340+3_340+10delACGGGCCCinsCG | 35 | － | － | － | 38 |
| 39 | 111 | Proband, F | c.340+5G>C | 35 | － | － | － | － |
| 40 | 112 | Proband, F | c.344A>G p.His115Arg | 34 | － | 44 | 44 | － |
| 41 | 113 | Proband, M | c.343delC p.His115Thrfs*44 | 26 | － | 36 | 36 | － |
| 42 | 114 | Proband, M | c.349T>G p.Trp117Gly | 36 | 44 | 44 | 40 | － |
|  | 115 | Son | c.349T>G p.Trp117Gly | 12 | 11 | － | － | － |
|  | 116 | Sister1 | c.349T>G p.Trp117Gly | － | － | － | 53 | － |
|  | 117 | Sister2 | Unknown^a^ | － | － | － | 46 | － |
|  | 118 | Daughter of Sister2 | c.349T>G p.Trp117Gly | － | － | － | － | － |
|  | 119 | Brother1 | Unknown^a^ | 47 | － | 59 | 59 | － |
|  | 120 | Brother2 | Unknown^a^ | 44 | 43 | 44 | 44 | － |
|  | 121 | Son of Brother2 | c.349T>G p.Trp117Gly | 12 | － | － | 18 | 18 |
| 43 | 122 | Proband, F | c.349T>A p.Trp117Arg | 19 | － | － | 26 | － |
|  | 123 | Father | c.349T>A p.Trp117Arg | 27 | － | 48 | 60 | － |
| 44 | 124 | Proband, M | c.350G>C p.Trp117Ser | 18 | － | 24 | 24 | － |
| 45 | 125 | Proband, M | c.388G>C p.Val130Leu | 30 | － | － | － | － |
| 46 | 126 | Proband, M | c.388G>C p.Val130Leu | 30 | － | 31 | 31 | 30 |
| 47 | 127 | Proband, F | c.402-428delATTATTTGTGCCATCTCTCAATGTTGA p.Glu134Aspfs*72 | 25 | 25 | 25 | 28 | － |
| 48 | 128 | Proband, M | c.404T>A p.Leu135* | 32 | － | 38 | 38 | 40 |
|  | 129 | Father | Unknown^a^ | － | － | 58 | － | － |
|  | 130 | Daughter | Unknown^a^ | 13 | － | － | － | － |
| 49 | 131 | Proband, F | c.407T>G p.Phe136Cys | － | 29 | 31 | 31 | 18 |
| 50 | 132 | Proband, F | c.433_437delCAGCC p.Gln145Tyrfs*27 | 26 | － | 45 | 45 | － |
|  | 133 | Brother1 | Unknown^b^ | 29 | － | 48 | 48 | － |
|  | 134 | Brother2 | Unknown^a^ | 27 | － | － | － | － |
|  | 135 | Father | Unknown^a^ | 25 | － | － | － | － |
| 51 | 136 | Proband, M | c.435_436insGG p.Pro146Glyfs*14 | 25 | － | 23 | 21 | － |
|  | 137 | Mother | Unknown^b^ | 32 | － | 38 | － | 38 |
| 52 | 138 | Proband, M | c.464-1G>C | 28 | － | 28 | 28 | － |
|  | 139 | Daughter | c.464-1G>C | － | － | － | － | － |
|  | 140 | Son | c.464-1G>C | － | － | － | － | － |
|  | 141 | Mother | c.464-1G>C | 66 | － | 69 | 66 | － |
|  | 142 | Sister1 | c.464-1G>C | 32 | － | 36 | 36 | － |
|  | 143 | Son of Sister1 | c.464-1G>C | － | － | － | － | － |
|  | 144 | Daughter of Sister1 | c.464-1G>C | － | － | － | － | － |
|  | 145 | Sister2 | c.464-1G>C | 39 | － | 39 | 39 | － |
|  | 146 | Son of Sister2 | c.464-1G>C | － | － | － | － | － |
| 53 | 147 | Proband, F | c.464-1G>A | 18 | － | － | － | － |
|  | 148 | Mother | Unknown^a^ | 17 | － | － | － | － |
| 54 | 149 | Proband, F | c.451A>T p.Ile151Phe | － | － | 39 | 39 | － |
| 55 | 150 | Proband, M | c.452T>G p.Ile151Ser | 14 | － | － | 17 | － |
|  | 151 | Father | c.452T>G p.Ile151Ser | 22 | － | 28 | － | － |
|  | 152 | Grandmother | Unknown^a^ | 43 | － | － | － | － |
| 56 | 153 | Proband, F | c.452T>A p.Ile151Asn | － | 26 | － | 31 | － |
| 57 | 154 | Proband, M | c.464_468delTGTAT p.Val155Aspfs*17 | － | 34 | 34 | 34 | － |
| 58 | 155 | Proband, M | c.480delG p.Glu160Aspfs*10 | － | 21 | 26 | 26 | － |
|  | 156 | Sister | c.480delG p.Glu160Aspfs*10 | 21 | － | － | 27 | － |
|  | 157 | Son of Sister | c.480delG p.Glu160Aspfs*10 | － | － | － | － | － |
| 59 | 158 | Proband, M | c.481C>T p.Arg161* | 29 | － | 29 | 32 | － |
| 60 | 159 | Proband, F | c.481C>T p.Arg161* | 39 | － | 39 | 39 | － |
| 61 | 160 | Proband, M | c.481C>T p.Arg161* | － | － | 44 | － | － |
| 62 | 161 | Proband, F | c.481C>T p.Arg161* | 28 | － | 41 | 41 | 41 |
|  | 162 | Daughter | c.481C>T p.Arg161* | 14 | － | － | 14 | － |
| 63 | 163 | Proband, F | c.482G>A p.Arg161Gln | 37 | － | 37 | 37 | 17 |
|  | 164 | Brother | c.482G>A p.Arg161Gln | 27 | 26 | － | － | 8 |
|  | 165 | Niece | c.482G>A p.Arg161Gln | － | － | － | － | － |
|  | 166 | Mother | Unknown^b^ | － | － | － | 56 | 56 |
| 64 | 167 | Proband, M | c.481C>T p.Arg161* | 23 | － | 46 | 46 | － |
| 65 | 168 | Proband, F | c.481C>T p.Arg161* | 36 | － | - | 34 | － |
| 66 | 169 | Proband, F | c.481C>T p.Arg161* | 20 | － | 23 | 23 | － |
| 67 | 170 | Proband, F | c.486C>G p.Cys162Trp | 30 | － | － | － | － |
|  | 171 | Father | Unknown^b^ | 40 | － | － | － | － |
| 68 | 172 | Proband, F | c.485G>A p.Cys162Tyr | 43 | － | 43 | 43 | － |
| 69 | 173 | Proband, M | c.486C>G p.Cys162Trp | － | － | 33 | 33 | － |
|  | 174 | Mother | Unknown^a^ | 30 | 54 | 50 | － | － |
|  | 175 | Sister | c.486C>G p.Cys162Trp | 28 | － | 35 | 35 | － |
|  | 176 | Niece | Unknown^b^ | 12 | － | － | － | － |
|  | 177 | Aunt | c.486C>G p.Cys162Trp | 37 | 45 | 53 | － | － |
|  | 178 | Son of Aunt | c.486C>G p.Cys162Trp | － | － | － | － | － |
| 70 | 179 | Proband, F | c.486C>A p.Cys162* | － | － | 44 | 28 | － |
|  | 180 | Daughter | c.486C>A p.Cys162* | － | 17 | － | 17 | － |
|  | 181 | Brother | Unknown^a^ | 17 | － | － | － | － |
|  | 182 | Father | Unknown^a^ | 49 | － | 56 | 56 | － |
|  | 183 | Uncle | Unknown^a^ | 32 | 30 | － | － | － |
| 71 | 184 | Proband, M | c.499C>T p.Arg167Trp | － | － | 27 | 24 | 24 |
|  | 185 | Mother | c.499C>T p.Arg167Trp | － | － | － | － | － |
| 72 | 186 | Proband, F | c.499C>T p.Arg167Trp | 37 | － | 37 | 37 | 39 |
|  | 187 | Father | c.499C>T p.Arg167Trp | 65 | － | 54 | 67 | － |
|  | 188 | Sister1 | Unknown^a^ | 22 | － | － | － | － |
|  | 189 | Sister2 | c.499C>T p.Arg167Trp | 39 | － | － | 36 | － |
|  | 190 | Sister3 | c.499C>T p.Arg167Trp | － | 34 | － | 32 | － |
| 73 | 191 | Proband, F | c.499C>T p.Arg167Trp | 26 | 13 | － | － | － |
|  | 192 | Mother | c.499C>T p.Arg167Trp | 38 | 25 | － | 52 | － |
| 74 | 193 | Proband, F | c.499C>T p.Arg167Trp | 16 | － | － | － | － |
|  | 194 | Mother | c.499C>T p.Arg167Trp | － | － | － | － | 36 |
| 75 | 195 | Proband, M | c.499C>T p.Arg167Trp | － | － | － | － | 32 |
| 76 | 196 | Proband, M | c.499C>T p.Arg167Trp | － | － | － | － | 26 |
|  | 197 | Father | Unknown^a^ | － | － | － | － | 64 |
|  | 198 | Uncle | Unknown^a^ | － | － | － | － | 53 |
|  | 199 | Son of Uncle | c.499C>T p.Arg167Trp | － | － | 36 | － | 36 |
| 77 | 200 | Proband, F | c.499C>T p.Arg167Trp | 25 | － | － | － | － |
|  | 201 | Mother | Unknown^b^ | 51 | － | － | 46 | 25 |
|  | 202 | Aunt1 | Unknown^a^ | 27 | － | － | － | － |
|  | 203 | Daughter of Aunt1 | Unknown^a^ | － | － | － | － | 31 |
|  | 204 | Grandson of Aunt1 | Unknown^b^ | － | － | － | － | 9 |
|  | 205 | Aunt2 | Unknown^a^ | 43 | － | 36 | 48 | － |
|  | 206 | Son of Aunt2 | Unknown^b^ | － | － | 21 | 21 | 16 |
| 78 | 207 | Proband, F | c.500G>A p.Arg167Gln | 35 | － | － | 49 | 49 |
| 79 | 208 | Proband, M | c.500G>A p.Arg167Gln | 29 | － | 46 | 49 | 46 |
|  | 209 | Mother | Unknown^a^ | 40 | － | － | － | － |
|  | 210 | Sister | Unknown^a^ | 50 | － | － | － | － |
|  | 211 | Nephew | Unknown^a^ | 15 | 27 | 25 | － | － |
|  | 212 | Daughter | Unknown^b^ | 24 | 14 | － | － | － |
| 80 | 213 | Proband, M | c.500G>A p.Arg167Gln | 52 | － | － | － | － |
|  | 214 | Son | c.500G>A p.Arg167Gln | － | 26 | － | － | 31 |
|  | 215 | Sister | c.500G>A p.Arg167Gln | － | － | － | － | 14 |
|  | 216 | Mother | Unknown^a^ | － | － | 56 | － | － |
| 81 | 217 | Proband, F | c.500G>A p.Arg167Gln | 24 | － | 32 | 33 | － |
|  | 218 | Brother | c.500G>A p.Arg167Gln | 20 | － | － | － | 26 |
|  | 219 | Mother | Unknown^b^ | 35 | － | － | － | － |
| 82 | 220 | Proband, M | c.500G>A p.Arg167Gln | － | － | 22 | － | 36 |
|  | 221 | Mother | Unknown^a^ | 45 | － | 49 | － | － |
|  | 222 | Sister | Unknown^b^ | － | 30 | 27 | － | － |
| 83 | 223 | Proband, F | c.500G>A p.Arg167Gln | － | － | － | － | 38 |
|  | 224 | Brother | c.500G>A p.Arg167Gln | － | － | 53 | － | － |
|  | 225 | Mother | Unknown^a^ | 52 | － | － | － | － |
| 84 | 226 | Proband, M | c.500G>C p.Arg167Pro | 24 | － | － | 26 | － |
| 85 | 227 | Proband, F | c.509T>A p.Val170Asp | 23 | － | 39 | 39 | － |
|  | 228 | Sister | Unknown^a^ | － | － | 36 | - | － |
| 86 | 229 | Proband, M | c.533T>G p.Leu178Arg | 39 | 57 | 57 | 57 | － |
|  | 230 | Sister | c.533T>G p.Leu178Arg | 40 | － | － | － | － |
|  | 231 | Brother1 | c.533T>G p.Leu178Arg | － | 25 | 54 | － | － |
|  | 232 | Brother2 | Unknown^a^ | 35 | － | － | － | － |
|  | 233 | Son of Brother2 | c.533T>G p.Leu178Arg | － | － | 30 | － | － |
| 87 | 234 | Proband, F | c.565-570delGAAGAC p.Glu189Hisfs*24 | － | － | 27 | 27 | － |
|  | 235 | Mother | Unknown^a^ | － | － | － | 38 | － |
| 88 | 236 | Proband, M | c.642+70C>A | 18 | 28 | 30 | 30 | － |
| 89 | 237 | Proband, F | c.642+70C>T | － | － | 46 | － | － |
| 90 | 238 | Proband, M | c.1－?_340+?del (Exon1 deletion) | － | － | 22 | 22 | 22 |
| 91 | 239 | Proband, M | c.1－?_340+?del (Exon1 deletion) | 25 | 14 | － | － | － |
|  | 240 | Sister | c.1－?_340+?del (Exon1 deletion) | － | － | － | 28 | － |
| 92 | 241 | Proband, F | c.1－?_340+?del (Exon1 deletion) | 39 | － | 39 | 36 | － |
| 93 | 242 | Proband, M | c.1－?_340+?del (Exon1 deletion) | － | 18 | 18 | 16 | － |
|  | 243 | Mother | Unknown^a^ | － | － | 43 | 43 | － |
| 94 | 244 | Proband, M | c.1－?_340+?del (Exon1 deletion) | 28 | 41 | 40 | 40 | 40 |
|  | 245 | Daughter | c.1－?_340+?del (Exon1 deletion) | － | － | － | － | － |
| 95 | 246 | Proband, F | c.1－?_340+?del (Exon1 deletion) | 18 | － | 31 | 31 | － |
|  | 247 | Daughter | c.1－?_340+?del (Exon1 deletion) | － | － | － | － | － |
|  | 248 | Sister | c.1－?_340+?del (Exon1 deletion) | － | － | 44 | － | － |
|  | 249 | Son of Sister | c.1－?_340+?del (Exon1 deletion) | － | － | － | － | － |
| 96 | 250 | Proband, M | c.1－?_340+?del (Exon1 deletion) | 23 | 17 | － | 19 | － |
|  | 251 | Mother | c.1－?_340+?del (Exon1 deletion) | 43 | 35 | 44 | 44 | － |
| 97 | 252 | Proband, M | c.1－?_340+?del (Exon1 deletion) | 46 | 46 | 46 | 46 | － |
| 98 | 253 | Proband, F | c.1－?_340+?del (Exon1 deletion) | 27 | － | 27 | 27 | － |
|  | 254 | Father | c.1－?_340+?del (Exon1 deletion) | － | － | － | － | － |
|  | 255 | Grandmother | Unknown^a^ | － | － | 60 | 27 | － |
| 99 | 256 | Proband, F | c.1－?_340+?del (Exon1 deletion) | 31 | － | 32 | 32 | － |
|  | 257 | Mother | Unknown^a^ | 29 | － | － | － | － |
|  | 258 | Uncle | Unknown^b^ | 28 | － | 50 | － | － |
| 100 | 259 | Proband, F | c.1－?_463+?del (Exon1, 2 deletion) | 28 | 24 | － | 28 | － |
|  | 260 | Son | c.1－?_463+?del (Exon1, 2 deletion) | － | － | － | － | － |
| 101 | 261 | Proband, M | c.(?_－213)_(*3705_?)del (Exon1, 2, 3 deletion) | 32 | － | 33 | 35 | － |
| 102 | 262 | Proband, F | c.341－?_463+?del (Exon2 deletion) | － | － | 29 | 29 | 29 |
| 103 | 263 | Proband, F | c.341－?_463+?del (Exon2 deletion) | 33 | － | 40 | 40 | － |
|  | 264 | Father | Unknown^a^ | － | － | 68 | － | － |
| 104 | 265 | Proband, M | c.341－?_463+?del (Exon2 deletion) | － | － | 48 | 48 | 48 |
|  | 266 | Son | Unknown^b^ | 19 | 27 | － | － | － |
|  | 267 | Brother1 | Unknown^b^ | 36 | 46 | － | － | － |
|  | 268 | Son of Brother1 | Unknown^b^ | － | 16 | － | － | － |
|  | 269 | Brother2 | Unknown^a^ | 30 | 20 | 41 | － | － |
|  | 270 | Son of Brother2 | Unknown^b^ | － | 16 | － | － | － |
| 105 | 271 | Proband, F | c.341－?_463+?del (Exon2 deletion) | 42 | － | － | 36 | － |
| 106 | 272 | Proband, F | c.341－?_463+?del (Exon2 deletion) | 51 | 49 | 51 | 51 | － |
|  | 273 | Daughter | Unknown^a^ | 13 | － | － | － | － |
|  | 274 | Father | Unknown^a^ | 30 | － | － | － | － |
| 107 | 275 | Proband, M | c.341－?_642+?del (Exon2, 3 deletion) | 37 | 31 | － | 37 | － |
|  | 276 | Father | c.341－?_642+?del (Exon2, 3 deletion) | 50 | － | － | － | － |
| 108 | 277 | Proband, M | c.341－?_642+?del (Exon2, 3 deletion) | 16 | 20 | 28 | － | － |
|  | 278 | Father | Unknown^a^ | 48 | － | － | － | － |
| 109 | 279 | Proband, M | c.341－?_642+?del (Exon2, 3 deletion) | 26 | － | 40 | 40 | － |
| 110 | 280 | Proband, M | c.341－?_642+?del (Exon2, 3 deletion) | 16 | 15 | － | 22 | － |
|  | 281 | Father | c.341－?_642+?del (Exon2, 3 deletion) | 25 | 29 | － | － | － |
| 111 | 282 | Proband, F | c.341－?_642+?del (Exon2, 3 deletion) | 31 | － | 32 | 32 | － |
| 112 | 283 | Proband, M | c.341－?_642+?del (Exon2, 3 deletion) | 16 | 24 | － | 22 | － |
|  | 284 | Mother | Unknown^b^ | 35 | － | － | 43 | － |
|  | 285 | Aunt | Unknown^b^ | 17 | 47 | － | － | － |
|  | 286 | Uncle | Unknown^b^ | 17 | 30 | － | － | － |
|  | 287 | Grandmother | Unknown^a^ | 30 | － | － | － | － |
| 113 | 288 | Proband, M | c.464－?_642+?del (Exon3 deletion) | 23 | 24 | 23 | 23 | － |
|  | 289 | Sister | c.464－?_642+?del (Exon3 deletion) | 30 | － | 30 | 30 | － |
| 114 | 290 | Proband, M | c.464－?_642+?del (Exon3 deletion) | 38 | 41 | 41 | 41 | － |
| 115 | 291 | Proband, M | c.464－?_642+?del (Exon3 deletion) | 27 | － | 31 | 31 | 31 |
| Abbreviations: CHB, central nervous system hemangioblastoma; RA, retinal angioma; RCC, retinal cell carcinoma; PCT, multiple pancreatic cysts or tumors; PHEO, pheochromocytoma; ^a^, death before diagnosis; ^b^, no DNA sample available. * Each patient with unknown *VHL* mutations was diagnosed by the clinical criteria and had at least one first degree relative who had been diagnosed with VHL disease by *VHL* gene testing before this study. | | | | | | | | |
